# Primary care physicians’ attitudes to the adoption of electronic medical records: a systematic review and evidence synthesis using the clinical adoption framework

**DOI:** 10.1186/s12911-018-0703-x

**Published:** 2018-11-13

**Authors:** Amy O’Donnell, Eileen Kaner, Caroline Shaw, Catherine Haighton

**Affiliations:** 1 Institute of Health & Society, Newcastle University, Baddiley-Clark Building, Richardson Road, Newcastle upon Tyne, NE2 4AX UK; 2 Department of Social Work, Education and Community Wellbeing, Faculty of Health and Life Sciences, Northumbria University, Newcastle upon Tyne, NE7 7XA UK

**Keywords:** Electronic health records, Primary health care, General practitioners, Clinical adoption framework

## Abstract

**Background:**

Recent decades have seen rapid growth in the implementation of Electronic Medical Records (EMRs) in healthcare settings in both developed regions as well as low and middle income countries. Yet despite substantial investment, the implementation of EMRs in some primary care systems has lagged behind other settings, with piecemeal adoption of EMR functionality by primary care physicians (PCPs) themselves. We aimed to review and synthesise international literature on the attitudes of PCPs to EMR adoption using the Clinical Adoption (CA) Framework.

**Methods:**

MEDLINE, PsycINFO, and EMBASE were searched from 1st January 1996 to 1st August 2017 for studies investigating PCP attitudes towards EMR adoption. Papers were screened by two independent reviewers, and eligible studies selected for further assessment. Findings were categorised against the CA Framework and the quality of studies assessed against one of three appropriate tools.

**Results:**

Out of 2263 potential articles, 33 were included, based in North and South America, Europe, Middle East and Hong Kong. Concerns about the accessibility, reliability and EMR utility exerted an adverse influence on PCPs’ attitudes to adoption. However many were positive about their potential to improve clinical productivity, patient safety and care quality. Younger, computer-literate PCPs, based in large/multi-group practices, were more likely to be positively inclined to EMR use than older physicians, less-skilled in technology use, based in solo practices. Adequate training, policies and procedures favourably impacted on PCPs’ views on EMR implementation. Financial factors were common system level influencers shaping EMR adoption, from start-up costs to the resources required by ongoing use.

**Conclusions:**

By using the CA Framework to synthesise the evidence, we identified a linked series of factors influencing PCPs attitudes to EMR adoption. Findings underline the need to involve end-users in future implementation programmes from the outset, to avoid the development of an EMR which is neither feasible nor acceptable for use in practice.

**Trial registration:**

PROSPERO CRD42016038790.

## Background

Recent decades have seen rapid growth in the implementation of Electronic Medical Records (EMRs) in healthcare settings [1]. By supporting the systematic collection and storage of patient data, the potential benefits of EMRs are manifold. They can help: increase completeness and minimise error in patient records [2]; improve the quality of healthcare, for example by supporting enhanced adherence to clinical guidelines [3]; and promote increased efficiency in clinical workflows by facilitating structured data sharing across organisational and geographic boundaries [4, 5]. Whilst traditionally their main function has been to support day-to-day clinical practice, EMR data offer a range of potential secondary uses, from supporting commissioning and healthcare planning [4, 6, 7], to helping patients have more control over their records [8, 9].

Effective implementation of EMRs in primary healthcare provides a unique opportunity to collect a wide-range of ecologically valid patient data to support understanding of disease burden and health trajectories over the life-course [10]. From the mid-1990s, there has been substantial investment in the information technology capabilities of primary care systems, particularly in developed regions such as Western Europe, North America and Australia [11, 12]. However examples of EMR initiatives are found increasingly in low and middle income countries (LMIC), such as Kenya and Brazil [13]. Indeed it has been suggested that by making more efficient use of resources, EMRs could help counter relative scarcity in the LMIC clinical workforce [14].

However, there are significant challenges associated with the introduction of EMRs: they are costly initiatives to implement, requiring time to tailor systems to suit local contexts, and to train end users [15]. This can prove a particular barrier for resource-poor settings, who may lack the qualified and experienced workforce to support their effective adoption [14]. Moreover, some of the benefits of EMRs have been disputed, particularly whether they have resulted in tangible improvements in the quality or efficiency of care [16–19]. Lack of interoperability between proprietary systems has limited the potential for efficient data-sharing, and patients and providers alike have expressed concerns about the security of personal health information stored in EMRs [20]. Such challenges may explain why, despite substantial investment, the implementation of EMRs in some primary care systems has lagged behind other settings [5, 12], with piecemeal adoption of EMR functionality by primary care physicians (PCPs) [21], especially those in small or solo practices [22].

**Fig. 1:**
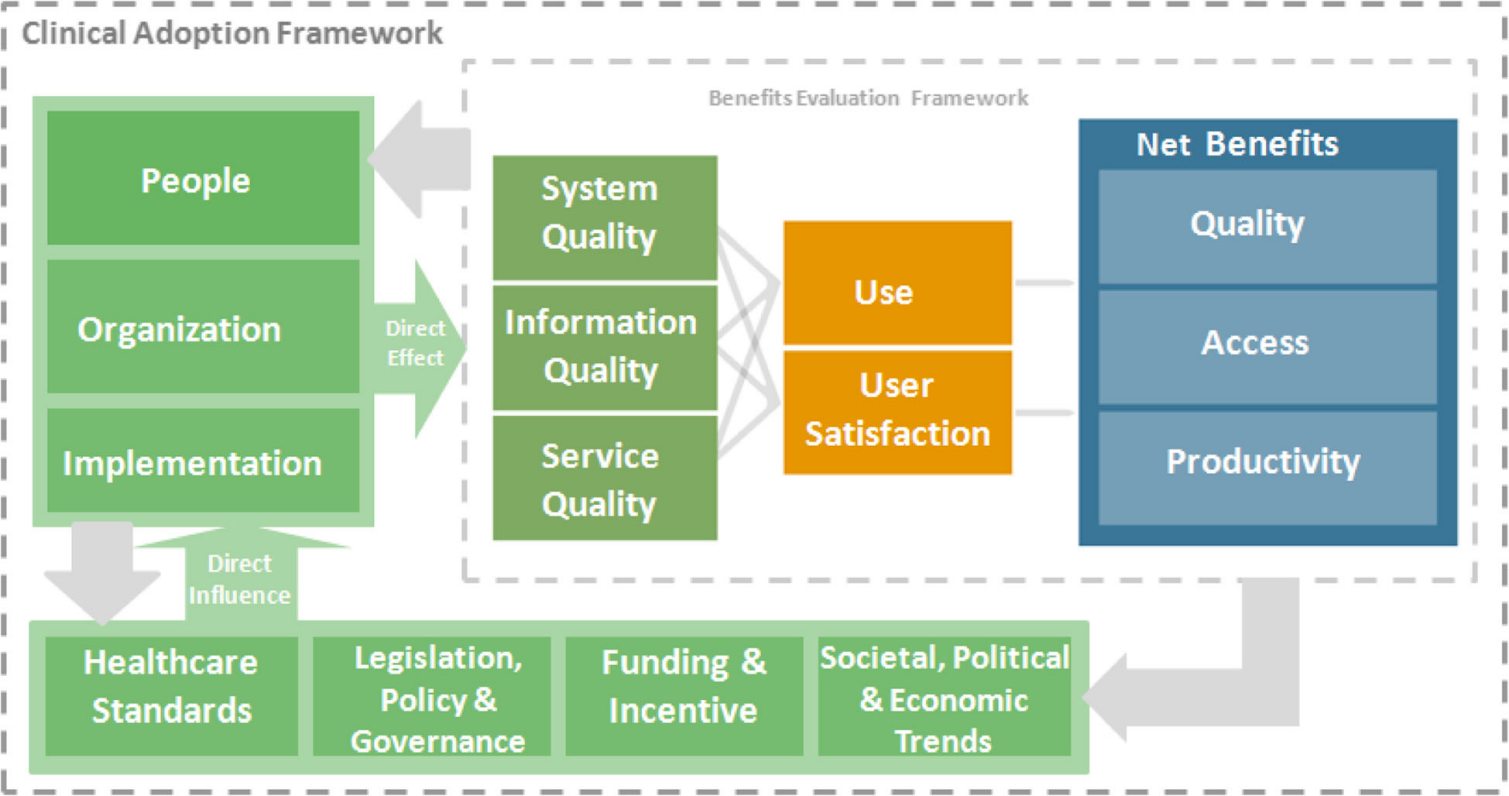
Clinical Adoption Model

To date, consideration of the structural factors shaping EMR implementation has limited the attention given to social and psychological influencers [20, 23]. However, as frontline users of EMRs, PCP attitudes have a significant impact on successful adoption [23, 24]. We aimed to review international literature on the attitudes of PCPs to EMR adoption in routine practice, using the Clinical Adoption (CA) Framework to guide categorisation and assessment of the evidence (Registration: PROSPERO CRD42016038790). The CA Framework consists of micro, meso and macro dimensions encompassing: the quality, accessibility and functionality of the EMR system; the people, organisation and process involved in EMR implementation; and the societal, political and legislative context [25, 26]. Figure 1 provides a conceptualisation of the framework in diagrammatic form, with further narrative describing the micro, meso and macro dimensions of the CAF outlined in Table 1.

**Table 1:** Micro, meso and macro level dimensions and categories of the Clinical Adoption Framework

Dimension	Category
Micro level	Health Information System (HIS) quality, which refers to the accuracy, completeness and availability of the clinical information content; features, performance and security of the system; and responsiveness of the support services.
	Usage quality which refers to HIS usage intention/pattern and user satisfaction in terms of usefulness, ease of use and competency.
	Net benefits, which refer to the change in care quality, access and productivity as a result of HIS adoption by clinicians. Care quality includes patient safety, appropriateness/ effectiveness, and health outcomes. Access refers to provider/patient participation and availability/access to services. Productivity covers care coordination, efficiency and net cost.
Meso level	People meaning the individuals/groups involved, their personal characteristics and expectations, and their roles and responsibilities with the HIS.
	Organization which refers to how the HIS fits with the organization’s strategy, culture, structure/processes, info−/infrastructure, and return on value.
	Implementation which involves the HIS adoption stages, project management approaches and the extent of the HIS’s fit for the practice.
Macro level	Healthcare standards in terms of the types of HISs organizational performance and professional practice standards in place.
	Funding and incentives which refer to the added values, remunerations and incentive programs.
	Legislation/policy and governance in terms of the influence of legislative acts, regulations/policies and governance bodies, such as professional associations/colleges and advocacy groups, and their attitudes toward HIS.
	Societal, political and economic trends which include public expectations and the overall socio-political and economic climates with regards to technologies healthcare and HIS.

## Methods

### Data sources and search strategy

We searched MEDLINE, PsycINFO and EMBASE on 1st August 2017 for papers published from 1st January 1996 to search date. Search terms encompassed four concepts: (1) PCPs; (2) primary care; (3) attitudes; (4) EMRs. Terms were coupled with relevant MeSH/thesaurus terms, truncated as appropriate, and variant spellings used. Bibliographies of related reviews, outputs of key journals and reference lists held by reviewers were hand-searched. Only published peer-reviewed articles were retrieved for further review.

### Inclusion criteria

EMRs were defined as computerised medical information systems that collect, store and display patient information [27]. The main user of the EMR had to be a medically qualified physician who provided primary healthcare, including general practitioners, family doctors, family physicians and family practitioners. Primary healthcare was defined as general healthcare covering a broad range of presenting problems, which can be accessed by a wide range of patients on demand, and not as the result of a referral for specialist care [28]. Studies needed to investigate attitudes towards the adoption of EMRs. We defined ‘attitude’ as a psychological disposition that is expressed by evaluating a particular entity favourably or unfavourably [29].

Outcome measures of interest were: (1) any reported measure of PCPs’ knowledge, attitudes or satisfaction with EMRs; (2) any objective or blind measure of EMR use (by standardised patient, other trained observer or video/audio recording). Papers not reporting either of these outcomes of interest were excluded, as were studies based on secondary data. Due to the rapid expansion of digital technology in healthcare from the mid-1990s, we restricted eligibility to studies published from 1996.

### Data selection and extraction

Search results were downloaded to EndNote version X7 and de-duplicated. Titles and abstracts of potentially relevant references were screened independently by two researchers (AOD, CS), who also reviewed full-texts of eligible papers. Disagreements were resolved by discussion with a third team member. A structured form guided data extraction of key study characteristics, including setting, participants, aim and methodology. Findings were mapped and categorised against the CA Framework (see Fig. 1).

### Quality assessment

Quantitative studies were quality assessed using the appropriate Center for Evidence-Based Management’s Critical Appraisal Checklist [30, 31]. Qualitative studies were assessed using the Critical Appraisal Skills Programme (CASP) Research Checklist [32]. Mixed-methods studies were assessed using a combination of these tools.

## Results

### Characteristics of the included studies

**Fig. 2:**
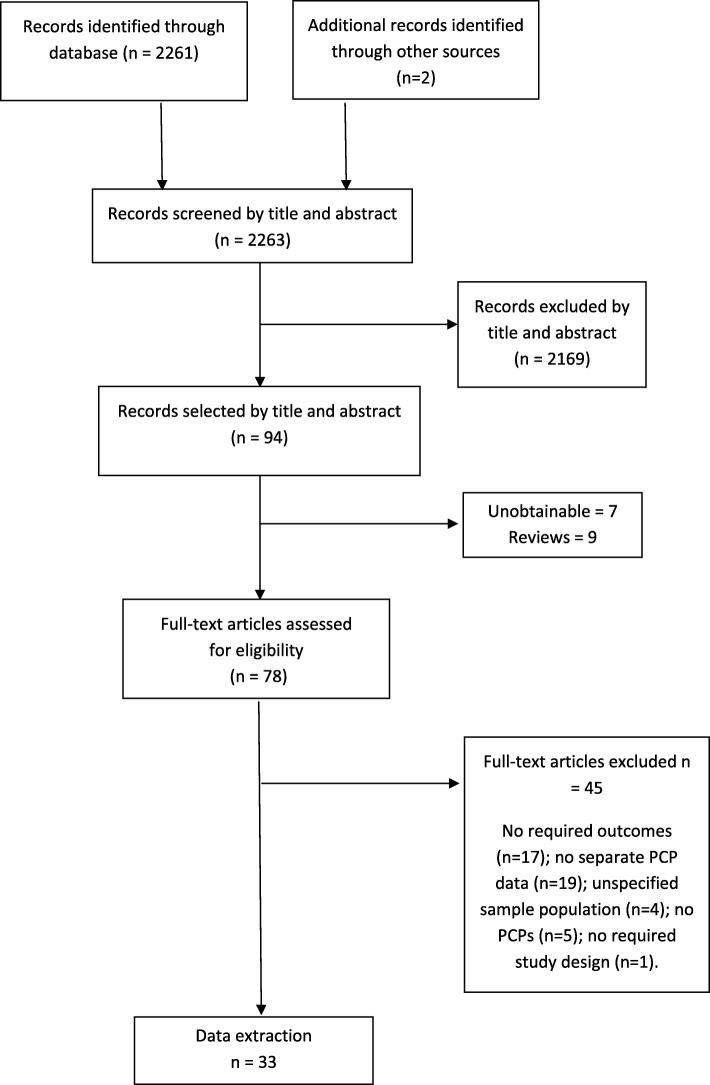
Flow chart showing the number of potentially relevant references identified by searches and number meeting inclusion criteria and included in the review

We identified 33 articles (see Fig. 2): 15 based in the USA [33–47]; 11 Europe [48–58]; two Canada [59, 60]; and one each in Saudi Arabia [61], Brazil [62], Hong Kong [63] and Israel [64]. The literature was methodologically diverse, including 11 qualitative studies [35, 36, 41, 44, 47, 49, 54, 59, 63–65], 17 cross-sectional surveys [33, 34, 37, 39, 42, 43, 45, 46, 50–52, 55–58, 60, 62], one longitudinal survey [53] and four mixed methods studies [38, 40, 48, 61]. Publication years ranged from 2001 to 2016. Of the 15 papers based on qualitative studies (including mixed methods studies with a qualitative component): one was deemed of low [61]; nine moderate [33, 35, 36, 41, 48, 49, 54, 64, 65]; and five high quality [44, 47, 59, 63]. Of the 20 papers based on cross-sectional studies (or a cross-sectional component): 14 were moderate [37, 38, 40, 42, 43, 45, 46, 48, 55–58, 60, 61] and six high quality [34, 39, 50–52, 62]. The final study employing cohort methods was categorised as moderate quality [53]. Full study details are available in Table 2.

**Table 2:** Summary of micro, meso and macro level factors influencing PCP attitudes to EMR adoption

Study	Design^a^ (Country)	Micro level			Meso level			Macro level			
		Quality	Use and user satisfaction	Net benefits	People	Organization	Implement-ation	Healthcare standards	Funding and incentives	Legislation/policy/governance	Societal/political/economic trends
Aaronson et al. (2001) [33]	SQ (USA)	NR	NR	NR	Neutral: Prior computer experience.	NR	Positive: Training length.	NR	NR	NR	NR
Alasmary et al. (2014) [61]	SQ + QI (Sauda Arabia)	NR	NR	Positive: Improved clinical productivity.	Positive: Computer literacy.	NR	NR	NR	NR	NR	NR
Christensen & Grimsmo (2008) [48]	FG + SQ (Norway)	Negative: Time-consuming navigation; Lack of accessible patient reports.	Negative: Impact on clinician-patient relationship.	Positive: Time-saving e.g. automated prescription renewal/key administrative and clinical information.	NR	Negative: Shifted administrative workload from health secretaries to PCPs.	NR	NR	NR	NR	NR
De Lusignan et al. (2003) [49]	QI (UK)	Negative: Challenging to locate appropriate Read Codes. Positive: Templates/lists of key codes helpful.	Negative: Challenging to record emerging diagnoses/vague symptoms; Risk of stigmatising patients.	Positive: Supported audit and feedback to demonstrate quality of care.	NR	NR	NR	NR	Positive: Financial support.	NR	NR
Desroches et al. (2013) [34]	SQ (USA)	Negative: Difficult to generate specific lists of patients.	Positive: Existing experience/meaningful EMR use.	NR	NR	NR	NR	NR	NR	NR	NR
Djalali et al. (2015) [50]	CSQ (Switzerland)	NR	Negative/neutral: impact on workflow processes; Impact on physician-patient-relationship	Negative/neutral: Impact on quality of care. Positive: impact on operating costs, time, cooperation and provision patient reports.	Positive: Younger, less clinicially experienced PCPs.	Positive PCPs based in a group practice compared to single-handed practice.	NR	NR	NR	NR	NR
Dossa & Welch (2015) [35]	QI (USA)	NR	Negative: Challenging to record sensitive information; Risk of stigmatising patients.	NR	NR	NR	NR	NR	NR	Positive: Availability of robust EMR privacy laws.	NR
Doyle et al. (2012) [36]	QI (USA)	Positive: Improved organization and accessibility.	Negative: Impact on patient interaction.	Positive: Impact on medication management.	NR	NR	NR	NR	NR	NR	NR
Emani et al. (2014) [37]	CSQ (USA)	NR	NR	Negative/neutral: Impact on medical errors; effectiveness/patient-centered/quality of care.	NR	NR	NR	NR	NR	NR	NR
Ernstmann et al. (2009) [51]	SQ (Germany)	Negative: System specification did not meet needs.	NR	Positive: Impact on medication errors; communication; administration time.	NR	NR	Positive: Training to improve system familiarity.	NR	NR	Positive: Belief that PCP interests were considered by policy makers/ represented by medical associations.	NR
Goetz Goldberg et al. (2012) [38]	SQ + QI (USA)	Negative: Difficult to navigate; Not customizable; Difficult to track patients; Disruptive impact of system failures.	Negative: Impact on patient interaction.	Positive: Impact on organization, accessibility, accuracy of patient data; Impact on communication; Potential to generate patient reports; Potential to support quality-improvement. Negative: Time-commitment.	NR	Positive: PCPs based in a group practice compared to single-handed practice.	Positive: PCPs based in practices that redesigned work processes, policies and procedures to support implementation	NR	Negative: Cost of upgrading system.	NR	NR
Greiver et al. (2011) [59]	FG (Canada)	Negative: Complex/ inflexible system; Software interface issues and immaturity; Adverse impacts of IT structural failures inc. lack of technical support.	Negative: Impact on patient interaction.	Negative: Reduced efficiency e.g. additional data entry time. Positive: Improved efficiency e.g. automated prescription renewals/consultation letters; Quality/accessibility of patient records.	Negative: Lack of basic IT/keyboard skills; Limited benefits for older PCPs.	NR	Negative: Lack of ongoing training post-implementation; Lack of technical support. Positive: Having designated champion to support/problem solve.	NR	Negative:Cost of system installation	NR	NR
Holanda et al. (2012) [62]	CSQ (Brazil)	Negative: Speed; Technical failures; Lack of functionality e.g. checking lab results. Negative/neutral: Accessibility of previous notes; Ability to review medication list.	NR	Negative: Speed in comparison to paper records.	Neutral: Length of clinical experience^;^.Positive: Basic computer literacy; Being female; Younger PCPs.	Positive: Seeing less than 16 patients per half-day.	NR	NR	NR	NR	NR
Keddie & Jones (2005) [52]	CSQ (UK)	Negative: Incompatibility with secondary care systems; Inability to transfer records between practices.	Negative: Intrusion of PC in consulting room; Lack of fit with current work practices.	Negative: Time-consuming.	NR	NR	Negative: Lack of training; lack of technical support.	NR	Negative: Cost of system installation.	Negative: Concerns about the medico-legal implications; Llack of policy-maker support for implementation.	NR
Loomis et al. (2002) [39]	CSQ (USA)	NR	NR	Positive: More secure and confidential than paper records.	Negative: Being a non-EMR user.	NR	NR	NR	NR	NR	NR
Meade et al. (2009) [53]	SQ (Ireland)	NR	NR	Negative: Time-consuming.	Negative: Lack of basic computer skills.	NR	Negative: Poor training.	NR	Negative: Cost of introducing system.	NR	NR
O’Malley et al. (2010) [65]	QI (USA)	Negative: Lack of system interoperability; Lengthy/ irrelevant problem lists.	Negative: Mismatch with work practices; Lack of usefulness for complex patients/situations.	Positive: Comprehensive/consistent/ accessible documentation; Automated record completion; Quality and efficiency of patient care.	NR	Negative: Limited impact on collaborative decision making.	NR	NR	Negative: Lack of financial and other incentives; Emphasis on use for billing and litigation prevention.	NR	NR
Or et al. (2014) [63]	QI + SQ (Hong Kong)	Positive: Accessible/efficient user-system interaction/interface; System flexibility and reliability.	Negative: Impact on patient interaction; Slower workflow.	Positive: Potential to improve medication management and/or patient safety issues. Negative: Burdensome data migration process and disruption to work processes	Negative: Lack of basic computer skills.	NR	Positive: Provision of post-implementation technical support and training.	NR	Negative: Cost of introducing system.	NR	NR
Pare et al. (2014) [60]	SQ (Canada)	Negative: Poor quality systems e.g. usability, security); Lack of system interoperability.	Negative: Adverse impact on doctor–patient relationship.	Negative: Costs greater than potential benefits.	Negative: Lack of basic computer skills.	NR	Negative: Lack of expertise in EMR systems; Transience of software vendors; Lack of technical support.	NR	NR	NR	NR
Pizziferri et al. (2005) [40]	SQ (USA)	NR	Negative: Reduced time spent with patients.	Positive: Improved quality, access, and communication of records.	NR	NR	NR	NR	NR	NR	NR
Pocetta et al. (2015) [54]	QI (Italy)	NR	NR	Positive: Improved effectiveness and efficiency eg via audit-and-feedback. Negative: Time-consuming, esp. recording lifestyle data.	NR	NR	NR	NR	Negative: Lack of financial incentives; Lack of professional recognition for the additional work involved.	NR	NR
Prazeres (2014) [55]	SQ (Portugal)	NR	Neutral: Impact on patient interaction; Length of consultation time.	NR	NR	NR	NR	NR	NR	NR	NR
Rose et al. (2005) [41]	FG (USA)	Negative: Difficult to navigate and access patient notes; Lack of available screen real estate/ cluttered screen. Positive: Use of screen contrast/ colour; Ability to customize.	Negative: Mismatch with existing workflow patterns.	NR	NR	NR	NR	NR	NR	NR	NR
Rosemann et al. (2010) [56]	SQ (Switzerland)	NR	Negative: Impact on patient interaction; Impact on doctor-patient relationship.	Negative: Cost-benefit ratio.	Positive: Younger PCPs	Positive: PCPs based in group practices.	NR	NR	NR	Negative: Concerns re data security law.	NR
Sequist et al. (2007) [42]	SQ (USA)	Negative: Technical limitations eg slow response time.	Negative: Impact on patient interaction.	Negative: Clinical productivity loss; Patient privacy/safety. Positive: Quality of care.	Positive: More clinical experience Negative: Lack of basic computer skills.	NR	Negative: Lack of technical support; Lack of training.	NR	NR	NR	NR
Shachak et al. (2009) [64]	QI (Israel)	Positive: Data-related comprehensiveness, organization, and readability.	Positive: Reduced cognitive load; Simple to use. Negative: Impact on patient interaction.	Positive: Automated review of patients’ medical histories/ test results; Provided clinical decision aids; Enhanced patient safety. Negative: Introduced new types of medical errors e.g. typos.	Positive: Advanced computer/ communication skills.	NR	NR	NR	NR	NR	NR
Steininger & Stiglbauer (2015) [57]	SQ (Austria)	NR	NR	Negative: Impact on patient privacy.	NR	NR	NR	NR	NR	NR	NR
Stream (2009) [43]	SQ (USA)	NR	NR	Negative: Productivity loss.	NR	Positive: Presence of students and residents in practice; Attitude of individual practices; Being based in group rather than solo practices.	NR	NR	Negative: Start-up financial costs, ongoing financial costs and training costs; Pay-for-performance and interest free loans. Positive: Grants and increased reimbursement.	NR	NR
Villalba-Mora et al. (2015) [58]	SQ (Spain)	NR	Positive: Availability of ePrescription/ patient management services e.g. appointments and referrals.	NR	Positive: Being female; Having basic computer skills; Use of internet outside the workplace.	NR	NR	NR	NR	NR	NR
Williams et al. (2011) [44]	QI (USA)	Negative: Accessing/ navigating family history information.	Positive: Helping to directing patient care; Building relationship/rapport.	Positive: Increase in practice efficiency.	NR	NR	NR	NR	NR	NR	NR
Wright & Marvel (2012) [45]	SQ (USA)	NR	NR	NR	Positive: Younger PCPs.	NR	NR	NR	NR	NR	NR
Yan et al. (2012) [46]	SQ (USA)	Negative: Technical limitations.	Negative: Adverse impact on -patient interaction.	Negative: Substantial productivity loss against limited direct benefits.	Negative: Older PCPs; Lack of EMR experience; Lack of computer skills.	Neutral: Practice size.	Negative: Training needs.	NR	Negative: Substantial financial costs.	Negative Lack of uniform industry EMR standards.	NR
Zhang et al. (2016) [47]	QI (USA)	Positive: Use of templates. Negative: Time consuming functions e.g. clinical reminders; Technical limitations e.g. slow user interface, lack of shortcuts; limited flexibility	Positive: Promoted patient engagement as viewing tool. Negative: Adverse impact on -patient interaction.	Negative: Productivity loss	NR	NR	NR	NR	NR	NR	NR

^a^Design: CSQ - cross-sectional survey questionnaire; FG - focus groups; QI - qualitative interviews; SQ - survey questionnaire

### Micro level

#### Quality of information, system and service

Over half of the papers discussed the impact that the quality of the EMR itself exerted on PCPs attitudes to adoption. Common factors ranged from the ease of access, efficiency and functionality of the system, to its technical reliability once in use [34, 36, 38, 41, 42, 44, 46–49, 51, 52, 59, 60, 62–65].

Three papers identified the user interface design, including ease of log-in, as an important factor influencing adoption: even if an EMR had advanced features, if the initial interface was challenging, PCPs were likely to reject it [47, 59, 63]. Several articles focussed on dissatisfaction with the speed of the EMR, sometimes relative to the perceived superior performance of paper-based records [38, 42, 47, 62]. Navigating the EMR to locate key patient information was sometimes viewed as time-consuming [41, 48]. System complexity [59], such as long lists from which to choose the appropriate code [49], was highlighted as problematic, alongside criticism of the numerous steps needed to complete a clinical transaction [38, 47]. However, templates were welcomed as a structured means of entering data, and memory joggers, such as lists of key codes, were also considered helpful [47, 49].

A perceived mismatch between EMR functionality and the needs of PCPs in practice was a common theme. EMRs were often viewed as lacking an easily accessible overview of key patient data, particularly family histories [34, 41, 44, 48, 62]. Problem lists were seen as overly long, containing redundant or irrelevant information [65]. One study highlighted the problems caused by the limited amount of available screen real estate (browser window size), which meant that immediate information needs could not always be accommodated within a single screen [41]. However one paper found PCPs were satisfied with EMRs in terms of data-related comprehensiveness, organization, and readability [64], and another suggested mostly positive impacts on the organisation and accessibility of patient data [36].

PCPs found it challenging to track patients through the health system via EMRs, either over time or across care boundaries [34, 38]. Lack of interoperability of primary care EMRs with secondary care IT systems, alongside an inability to transfer electronic records between practices, were also highlighted as barriers to adoption [52, 60]. It was challenging to convert third-party diagnostic results into searchable structured data, with practices usually reliant on scanning paper documents into the EMR as PDF files [65]. Several papers focussed on system reliability as a barrier to adoption [38, 59, 60, 62], emphasising the major disruption to office operations and patient care caused by even occasional EMR system or server crashes [38, 62], often exacerbated by the lack of technical support available to practices [38, 59, 60]. Thus post-implementation technical support was important to support effective EMR adoption over time [63].

#### Use and user satisfaction

PCPs’ satisfaction with EMRs in routine practice was the most frequently reported influencing factor, mentioned by 22 papers [34–36, 38, 40–42, 44, 46–50, 52, 55, 56, 58–60, 63–65]. Two reported on incompatibilities between rigid EMRs and the complex, dynamic, medical decision-making process [41, 65]. Concerns were raised in nine about the potential negative influence of computers on the doctor-patient relationship [36, 46–48, 50, 56, 59, 60, 64], some highlighting the intrusive nature of having the computer screen in the consulting room [52], alongside the pressure of data entry, which could distract attention from the patient [36, 38, 40, 42, 48, 55, 63]. However, two papers suggested that PCP IT skills, especially blind typing and the use of keyboard shortcuts and templates, could help mitigate these issues [63, 64]. Moreover, if used to involve patients in their care, by screen-sharing or using elements as a visual learning tool, two papers identified the potential for EMRs to boost engagement [44, 47].

Several papers highlighted the difficulties experienced when using EMRs to record certain types of patient data [35, 49, 57]. One study reported that PCPs found it challenging to record emerging diagnoses and/or vague symptoms, particularly when a diagnosis was potentially sensitive or stigmatising [49]. It was suggested that certain information was sometimes excluded from the EMR out of respect for the patient’s wishes or because of the potential consequences for the patient if external agencies accessed the information [35]. Another study emphasised how recording behaviour was shaped by PCPs’ perception of the value of information recorded [54]. For example, smoking was most frequently recorded and updated because it was used to help calculate an individual’s cardiovascular risk score. The recording of alcohol consumption, physical activity and eating habits was far less routine [54].

Four papers discussed the positive influence of long term EMR use on adoption attitudes [34, 39, 44, 58]. Users were more likely than non-users to believe that current EMRs were helpful [39, 58]. PCPs working in practices with even a relatively basic system were significantly more likely than those without to hold positive opinions about increased EMR use [34]. Positive user experiences were also related to the perceived usefulness of the information gathered via the EMR, such as helping to direct patient care based on family histories or for building rapport [44].

#### Net benefits in terms of care quality, productivity and access

Twenty five papers discussed PCPs’ perceived (dis)benefits of EMR adoption across a range of areas [34, 36, 38–40, 42–44, 46–54, 56, 57, 59–65]. A number of studies focussed on their potential to improve clinical productivity [36, 38–40, 44, 48, 50–54, 59–65], for example by reducing the coding mistakes commonly encountered in paper-based records [61]. Anticipated gains also resulted from the automation of key clinical functions, such as prescription renewals and issuing referral letters [48, 59, 65]. Increased efficiency due to the improved accessibility of patient data was a common theme [36, 38, 40, 54, 59, 64], with PCPs in two studies thinking that EMRs would eliminate the problems caused by poor organization of paper charts and illegible handwriting [36, 64].

The positive impact of EMRs on practice communication was reported in five papers [38, 40, 50, 51, 65]. Some PCPs highlighted use of the patient problem list, task assignment functions, and to-do lists as communication tools between clinicians, administrators, and patients [38]. For others, EMRs enabled improved coordination within the practice by making patient notes more legible, organized and retrievable [65]. Users were also more likely than non-users to view systems as improving the security and confidentiality of patient records [39].

Patient safety and care quality were two further areas perceived as benefiting from EMR introduction [38–40, 49, 65], particularly by more experienced users [39]. The potential of EMRs to support audit and feedback was an important motivating factor for adoption [49, 54], as was the ability to generate reports for quality-improvement purposes [38]. PCPs in a number of studies anticipated that the adoption of EMRs would result in improved medication management [36, 51, 63–65]. By enabling more accurate, comprehensive, and automated documentation of medications [63, 65], EMRs could help to: identify and flag drug interactions [64]; identify patients affected by a drug recall; and manage prescriptions for controlled drugs more effectively [36].

Focussing on perceived disbenefits of EMR adoption, the time [38, 53, 59, 63] and cost [43, 46, 52, 56, 60, 63] associated with installing a new electronic system were flagged. Three studies drew attention to the additional time required to enter patient data in EMRs [38, 47, 59], which reduced PCPs’ time for patient care. The initial capital outlay required for EMR implementation was highlighted as a barrier by several papers; most suggested that PCPs felt unlikely to benefit directly from such investment due to the increased workload associated with using the system [46, 56, 60, 63]. Start-up costs emerged as the main barrier, but the ongoing financial commitment of maintaining EMRs, including training practice staff, was also an obstacle highlighted by PCPs [43, 52, 63].

Contradictions emerged with regards to the perceived benefits of EMRs reported above. PCPs in three studies were either neutral or negative about their impacts on care quality [37, 42, 50], and a small number of respondents in one study raised concerns about patient privacy and safety [37]. Less than half of PCPs’ believed that EMRs would reduce errors in medical records in one paper [37]. Another raised concerns that EMR use could actually introduce new types of medical errors, including typos, adding information to the wrong patient’s chart, and unintentionally selecting an erroneous item (diagnosis or medication) from scroll-down lists [64].

### Meso level

#### People

Fifteen papers described PCP characteristics potentially associated with attitudes to EMR adoption [33, 39, 41, 42, 45, 46, 50, 53, 58–64]. Three highlighted gender as a significant influencer, although in one, male PCPs demonstrated higher levels of EMR adoption [50], whereas the opposite was true for the remainder [58, 62]. Age was a commonly occurring factor. Four papers suggested that younger PCPs were more likely to adopt EMRs than older PCPs [46, 50, 56, 62], and another highlighted a perception that such increased adoption rates were because the benefits of EMRS were greater for younger PCPs [59]. One study found that older PCPs were more likely to express satisfaction with EMR use [62], but this contrasted with findings elsewhere [45].

Several papers highlighted PCPs’ lack of basic computing and keyboard skills as a substantial barrier to the level [42, 53, 59, 60] and quality [64] of EMR adoption. Three suggested that PCPs with prior computing experience were more likely to demonstrate positive attitudes towards adoption and regular use [58, 61, 62], with non-users much less positive [39]. However two further studies found computing and/or EMR experience to be a relatively neutral [33] or infrequently mentioned influencing factor [46]. In terms of broader clinical experience, one study found that PCPs with fewer years in medical practice appeared more positively inclined to EMR use [50], although another [62] found this relationship weak, and a further study suggested that more clinically experienced PCPs were actually higher adopters [42].

#### Organization

Practice characteristics also influenced adoption attitudes [38, 43, 46, 48, 50, 56, 62, 65], particularly size [38, 43, 50, 56], with adoption higher in large [50] and group practices [40, 43, 56]. Advanced EMR use (i.e. using data for monitoring and evaluation purposes) was more often seen in practices owned by large, healthcare organisations, in part due to availability of technical and administrative support [38]. PCPs working in single-handed practices were more likely not to have any computer or to have computers for reception purposes only, which impacted negatively on their attitudes towards EMR adoption [43, 46, 50]. Only one study found that practice size was not associated with PCPs’ barriers to EMR adoption [46]. However findings from another suggested that individual practices had more impact on EMR adoption than larger practice networks [43].

Additional organisational factors shaping adoption concerned the alignment of EMRs within existing working practices, roles and responsibilities, and patient throughput; although here evidence was conflicting [46, 62]. A final study highlighted a perception that EMR introduction led to a shift in administrative workload from health secretaries to PCPs [48].

#### Implementation

The EMR implementation process influenced PCP views in ten studies [33, 38, 42, 46, 51–53, 59, 60, 63], with training the most frequently mentioned factor. Lengthier training provision impacted positively on both PCPs’ views on implementation and their ongoing use of the system [33]. Three papers reported that inadequate training was an important barrier to EMR use [42, 46, 53]; another highlighted the need for ongoing training post-implementation [59]. Lack of expertise, time and knowledge to manage the implementation process was also a barrier to adoption [60]. Practices that experienced smoother transitions to EMR use had redesigned work processes and developed policies and procedures to support implementation [38]. Finally, having a designated EMR champion could facilitate improved EMR adoption [59].

### Macro level influencers

#### Funding and incentives

Financial factors shaped PCP’s adoption attitudes in ten studies [38, 39, 43, 46, 49, 52–54, 59, 63, 65]. The costs associated with purchasing the technology [38, 53, 59] alongside additional human resource demands, were highlighted as key barriers to adoption [59]. Lack of financial incentives resulted in more limited, less integrated use of EMRs [65]; one paper suggested this was construed as a lack of priority for both the work involved in implementing EMRs and the data collected [54]. Several suggested that provision of grants and reimbursement schemes for EMRs would result in more effective implementation [43, 63]. However, sustained use over time increased PCPs willingness to invest in EMRs [39].

#### Legislation, policy and governance

Six studies flagged policy and legislative influences [35, 46, 51, 52, 54, 56]. Concerns around data security were highlighted [52, 56], although one paper suggested that availability of robust privacy laws could facilitate more comprehensive EMR use [35]. Evidence was inconsistent regarding the extent to which PCPs felt supported by policy makers in either the design or implementation of EMRs [51, 52].

No papers explicitly identified healthcare standards or societal, political and economic trends as macro level factors influencing adoption.

## Discussion

This systematic review found that multiple and interrelated influencers shape PCPs’ attitudes towards EMR implementation in routine practice, although several common themes emerged across the literature. As highlighted in previous reviews [18, 23, 24, 66–68], concerns about the accessibility, reliability and overall utility of the EMR appear to exert a sizeable adverse influence on PCPs’ attitudes to adoption. Like Boonstra and Broekhuis [23], we found that PCPs perceived a mismatch between the rigid functionality of the EMR and their more complex, dynamic needs as family physicians, which negatively affected productivity. Lack of EMR interoperability, limiting physicians’ ability to exchange electronic information between other general practices or with secondary care IT systems, was also highlighted as a barrier; again a strong theme emerging in other evidence syntheses [23, 24, 27, 68, 69]. At the same time, as Audet et al. found [22], many PCPs were positive about EMRs’ potential to improve clinical productivity, and valued the automation of key clinical functions, like prescription renewals. Echoing Nguyen et al’s evaluation [68], there was also a perception that patient safety and care quality could benefit from EMR introduction, by supporting the audit-and-feedback process, alongside improved medication management. However other reviews have found less conclusive evidence for this point [66, 70].

Certain PCP characteristics themselves appeared to be associated with views on EMR adoption. Younger PCPs, with an appropriate level of computing skills, and based in larger or multi-group practices, were more likely to be positively inclined towards using EMRs than older, physicians, less-skilled in technology use, and working in solo practices [see also 22, 66 on this point]. Like Castillo et al. amongst others [20, 23, 24, 27], we found that efforts made to implement EMRs, including adequate training provision both during and post-implementation, up-to-date policies and procedures, and the presence of a designated EMR champion, had a favourable impact on both PCPs’ views on EMR adoption, and their ongoing use of the system.

Financial factors were the most common system level dimension shaping PCP’s adoption of EMRs, from the initial costs of purchasing the technology, to the additional demands required by their ongoing use: an issue emphasised in numerous existing reviews [23, 27, 65, 66, 68, 69]. A lack of financial incentives or reimbursements to support installation resulted in more limited, less integrated EMR use by PCPs. However this was not a uniform finding, with other studies finding ambiguous evidence for the effectiveness of financial incentives on EMR adoption [71]. There was mixed evidence on the extent to which PCPs felt supported and represented by policy decisions linked to the design and implementation of EMR systems. Ross et al. stress the adverse impact that absent or inadequate policy and legislation can have on the adoption of e-health systems in general [69]. Finally, whilst concerns around data security were mentioned, in contrast to other studies [23, 66], this was a relatively minor theme overall in our review.

**Fig. 3:**
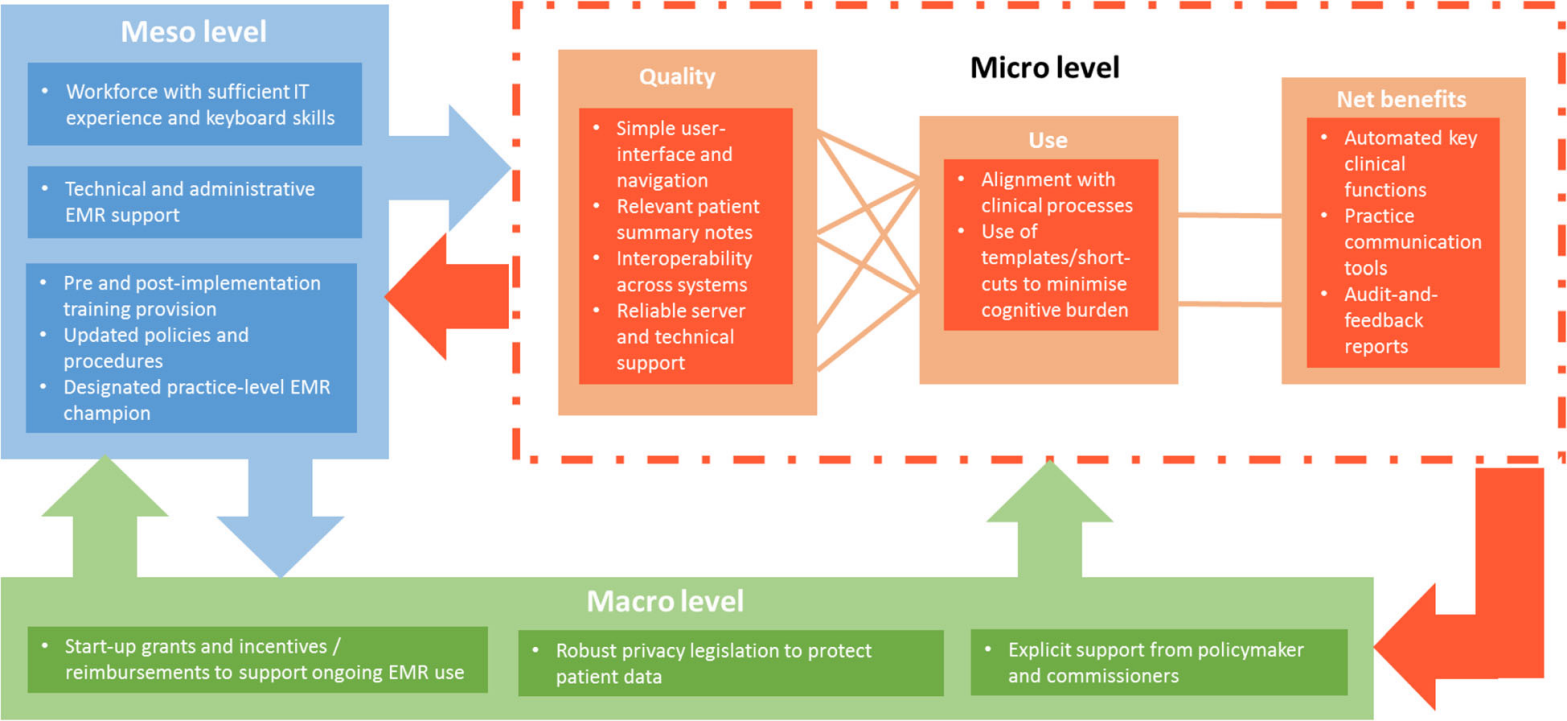
CA Framework of micro, meso and macro factors facilitating positive PCP attitudes to EMR adoption

Our findings serve to highlight that EMR programmes are complex interventions which must be implemented in dynamic socio-technical health systems [72]. Figure 3 illustrates the key features a successful plan to boost EMR adoption should include based on our results .

However, as Lau et al. stress [73], whilst these features may be conceptualised at either the micro, meso or micro level, in reality, they operate interdependently. Thus an effective EMR programme needs to take into account not just these features in static isolation, but also consider how they interact and evolve over time [74]. Further, with accelerating adoption of electronic records across health and social care settings [68], and increasingly complex population health needs [75], there is a real need to improve interoperability across systems, to ensure that patients receive coordinated care of consistent quality [76]. Various obstacles exist to realizing this aim, from how we can allay patient and practitioners’ valid concerns around the ethics of personal data sharing, to the challenge of overcoming more technical and semantic hurdles. Whilst progress is being made towards building data systems that permit functional interoperability [77], sharing and using data effectively will also require the adoption of common standards, ontologies and terminologies across multiple sectors and institutions [78].

Ultimately, however, EMR adoption is determined by the attitudes and behaviours of the individual clinician themselves [24]. Decades of research emphasises the positive effects of early user involvement in design and development on the eventual success of implementing new technology [19]. Yet a dominant theme in this review was a perceived lack of fit between proprietary EMR systems, and the values, priorities and work practices of PCPs themselves [79–81]. For example, the adverse impact of EMR adoption on the doctor-patient relationship was an enduring concern that was highlighted across the period covered by the included literature [36, 38, 40, 42, 44, 46, 48–50, 52, 55, 56, 59, 60, 63–65]. Future implementation programmes must provide a forum for end-users to play an active role in the design process from the outset, and consider the socio-technical “connectives” between clinician, system and patient [82]. This would help to boost a sense of psychological ownership of new systems [83], resulting in greater support for technological change [84], as well as harnessing their expertise and experience, thereby avoiding the development of an EMR which is neither feasible nor acceptable for use [85, 86].

### Limitations

The systematic review methodology, a moderately sized and geographically varied evidence-base of 33 studies, were strengths of this study. However, our review was restricted to peer-reviewed papers, which could introduce publication bias [87]. Additionally, as highlighted in previous reviews [23, 66], the literature in this field can be poorly referenced within bibliographic databases, due to nonstandard use of terminology and lack of consensus on a taxonomy relating to e-health technologies [88].

We were also limited by the shortcomings of the literature based on methodological rigor, with the majority of included studies deemed of only moderate quality. Around half of the studies (17 out of 33) were structured surveys, using closed-ended questions to capture data, which inevitably limited respondents’ opportunities to highlight any issues not already prescribed in the questionnaire. Greenhalgh has written extensively about the need to move beyond positivist methodologies when evaluating eHealth programs [89]. Her framework for the evaluation of the introduction of shared electronic summary records in England incorporates social, technical, ethical and political dimensions [90]. However, there was limited data on macro or system level influencers, including policy and legislation, despite the fact that implementation science emphasises the importance of contextual factors in shaping adoption of new interventions and technologies [91].

The use of an alternative framework may have altered data synthesis and thus influenced findings. There are a number of potential theories, models and frameworks available to help understand EMR adoption, from broader implementation theories such as Roger’s classic Diffusion of Innovations [92], or the Consolidated Framework for Implementation Research [69], to more specific models like the Technology Acceptance Model (TAM) [67, 93–96], and new taxonomies developed by Boonstra and Broekhuis [23] or Castillo et al. [24]. In contrast to previous approaches however, the CA Framework provides a conceptual model to help describe the factors shaping EMR adoption which has been specifically contextualised to the healthcare setting, which also allows us to capture influencing factors operating at the organisational level and beyond [73, 97].

Evidence exploring the patient perspective on EMR use was outside the scope of this review, including the acceptability of patient-held medical records. Yet previous research emphasises that adoption of these two systems are inextricably linked [98]. Whilst our focus on PCPs was deliberate, it may limit the generalizability of our findings beyond primary healthcare [20, 99], as effective implementation of a comprehensive, system-wide EMR, would need to take account of the norms, values, and work practices of the full socio-technical network involved [90].

## Conclusions

By using the CA Framework to synthesise the evidence base, we have identified a linked series of factors influencing PCPs attitudes to the adoption of EMRs, which could usefully inform future implementation initiatives. Policymakers and system architects designing such initiatives need to recognise that EMR programmes are complex interventions, which must be implemented in dynamic social-technical systems, but that adoption is ultimately determined by the attitudes and preferences of the individual clinician.

## Abbreviations

CA Framework: Clinical Adoption Framework
CASP: Critical Appraisal Skills Programme
EMR: Electronic Medical Record
LMIC: Low and middle income country
PCP: Primary care physician
